# Biocidal activity of metalloacid-coated surfaces against multidrug-resistant microorganisms

**DOI:** 10.1186/2047-2994-1-35

**Published:** 2012-11-14

**Authors:** Nathalie Tétault, Houssein Gbaguidi-Haore, Xavier Bertrand, Roland Quentin, Nathalie van der Mee-Marquet

**Affiliations:** 1 Service de Bactériologie et Hygiène, Centre Hospitalier Universitaire de Tours, Tours, F37044, France; 2 Service d’Hygiène Hospitalière, Centre Hospitalier Universitaire de Besançon, Besançon, F25030, France; 3 Réseau des Hygiénistes de la région Centre, Hôpital Trousseau, Centre Hospitalier Universitaire de Tours, Tours, F37044, France

**Keywords:** Metalloacid-coated surface, Biocidal effect, Infection control

## Abstract

**Background:**

The antimicrobial effects of a coating of molybdenum trioxide (MoO_3_) has been recently described. The metalloacid material produces oxonium ions (H_3_O^+^), which creates an acidic pH that is an effective, non specific antimicrobial. We determined the *in vitro* antimicrobial activity of molybdenum trioxide metalloacid-coated surfaces.

**Methods:**

Metalloacid-coated and non-coated (control) surfaces were contaminated by exposing them for 15 minutes to microbial suspensions containing 10^5^ cfu/mL. Eleven microorganisms responsible for nosocomial infections were tested: two *Staphylococcus aureus* strains (the hetero-vancomycin intermediate MRSA Mu50 strain and a ST80-PVL-producing MRSA strain); a vancomycin-resistant *van*A *Enterococcus faecium* strain; three extended-spectrum beta-lactamase-producing *Enterobacteriaceae* strains; a MBL-producing *Pseudomonas aeruginosa* strain; a multidrug-resistant *Acinetobacter baumannii* strain; a toxin-producing *Clostridium difficile* strain; and two fungi (*Candida albicans* and *Aspergillus fumigatus*). The assay tested the ability of the coated surfaces to kill microorganisms.

**Results:**

Against all non-sporulating microorganisms tested, metalloacid-coated surfaces exhibited significant antimicrobial activity relative to that of the control surfaces within two to six hours after contact with the microorganisms (p < 0.001). Microorganism survival on the coated surfaces was greatly impaired, whereas microorganism survival on control surfaces remained substantial.

**Conclusions:**

We suggest that, facing the continuing shedding of microorganisms in the vicinity of colonized or infected patients, the continuous biocidal effect of hydroxonium oxides against multidrug-resistant microorganisms may help limit environmental contamination between consecutive cleaning procedures.

## Background

Nosocomial infections are a major cause of patient morbidity and mortality. Those associated with contaminated surfaces and the inadequate hand hygiene of healthcare workers (HCWs) are avoidable by cleaning and/or disinfecting environmental surfaces and by appropriate hygiene practices [1-4]. The transmission of microorganisms responsible for contaminated surface-associated nosocomial infections is well understood. Briefly, patients and HCWs carry and shed microorganisms around them. Surviving microorganisms contaminate near-patient items, which then act as reservoirs for microbial pathogens and become sources of contamination for the hands of HCWs and of colonization for new patients. Microbial colonization of surfaces can be restricted by cleaning procedures that employ effective chemical products. Cleaning reduces the prevalence of microorganisms in the environment and has been demonstrated to be a key-measure in the control of outbreaks associated with methicillin-resistant *S*. *aureus* (MRSA), vancomycin-resistant enterococci (VRE) and *A*. *baumannii*[1-7].

In fact, the continuing shedding of microorganisms between consecutive cleansing interventions, and the difficulties of cleaning particular medical devices, frequently result in residual environmental contamination. An approach to reducing the levels of microbial contamination on inanimate surfaces consists of using anti-adhesive coatings to prevent microorganism adhesion [8,9]. A second approach is to coat the surfaces of materials with disinfectants or with inorganic antimicrobials such as silver or copper ions [10,11]. Currently being used for catheters and dressings [12], these technologies are now being frequently applied to other types of medical equipment. The rapid release of the adsorbed biocide after implantation [13], cytotoxicity on mammalian cells [14] and the emergence of resistant microorganisms [15] may limit the biomedical device applications of these technologies. Zollfrank *el al*. recently reported the antimicrobial effects of a newly developed antimicrobial coating of molybdenum trioxide (MoO_3_) against two reference bacterial strains, *S*. *aureus* ATCC 25923 and *Pseudomonas aeruginosa* ATCC 10145 [16]. The metalloacid material produces oxonium ions (H_3_O^+^), which create an acidic pH (pH 5) that is an effective antimicrobial [17]. To assess the value of such coatings in preventing the generation of environmental microbial reservoirs on medical equipment and in diminishing the risk of exposing patients to microorganisms, we studied the biocidal activity of surfaces coated with the MoO_3_ metalloacid material. We tested eight multidrug-resistant bacteria strains representative of those that cause nosocomial infections and outbreaks worldwide, a spore-producing, toxin-producing strain of *Clostridium difficile*, and two fungi.

## Methods

### Microbial strains

Nine bacterial strains were used for the assay: two MRSA strains (the Mu50 reference strain and a ST80-PVL-producing strain); a vancomycin-resistant *van*A *Enterococcus faecium* strain; a toxin-producing *Clostridium difficile* strain, three extended-spectrum beta-lactamase (ESBL)-producing *Enterobacteriaceae* strains (*Escherichia coli*, *Klebsiella pneumoniae* and *Enterobacter cloacae*); a MBL-producing *Pseudomonas aeruginosa* strain and a MDR *Acinetobacter baumannii* strain. All strains except the Mu50 strain were isolated from nosocomial outbreaks in French healthcare institutions during the 12-month period preceding the study. We also tested two fungi: *Candida albicans* and *Aspergillus fumigatus*.

### Wires

Metalloacid-coated and non-coated EKG leadwires were cut into sections 2 cm long. Groups of six sections of coated and non-coated leadwires were placed into sterile boxes. Before each microbial contamination, the prepacked wires were pasteurized at 65°C for one hour.

### Contamination of leadwires

Microbial suspensions were prepared as follows: one colony of a fresh culture on an agar plate was used to inoculate brain-heart infusion broth. After incubation for 24 hours at 37°C, the final microbial suspension was prepared by adding 50 μL of the cultured broth to 100 mL of sterile saline solution. Ten mL of each microbial suspension was added to one test box containing the coated leadwire sections and one control box containing non-coated leadwire sections. The boxes were hermetically sealed and then agitated gently for 15 minutes at ambient temperature. The leadwire sections were then transferred to sterile tubes (one per tube) and this was defined as time 0 (t0). The samples were stored at 37°C for various times.

### Semi-quantitative assessment of microorganism survival

In account of the form of the leadwire sections used for the study, we applied the semiquantative culture method of Maki that is well-recognized method for evaluating the microbial density colonization on catheter surface [18]. At t0, and after two, four, six, 24 and 48 hours of storage at 37°C, Maki’s technique was performed by transferring each contaminated leadwire section to a blood agar plate and by rolling the section back and forth across the surface (one section per plate). The plates were then incubated for 48 hours at 37°C. After incubation, the plates were photographed and colonies were counted. For each of the tested microorganisms, the overall assay was repeated five times, and the bacterial counts were used to calculate average counts.

### Statistical analysis

Wilcoxon signed-rank tests were used to compare continuous paired data. For all calculations, a two-tailed *P* value of less than 0.05 was considered to indicate statistical significance. The software package Stata, version 10.0 (Stata Corp., College Station, TX, USA) was used for analysis.

## Results and discussion

**Figure 1:**
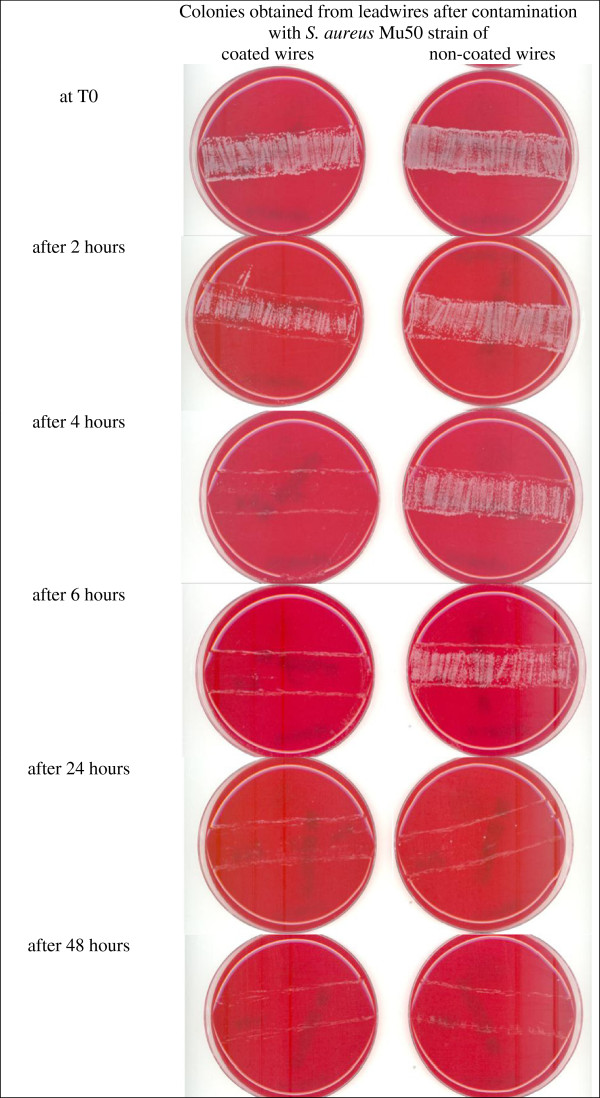
**Colonies obtained from contaminated leadwires.** At t0 and after 2, 4, 6, 24 and 48 hours of storage at 37°C, coated and non-coated leadwire sections contaminated with *Mu50 Staphylococcus aureus* were rolled on blood agar plates and then incubated for 48 hours at 37°C.

**Figure 2:**
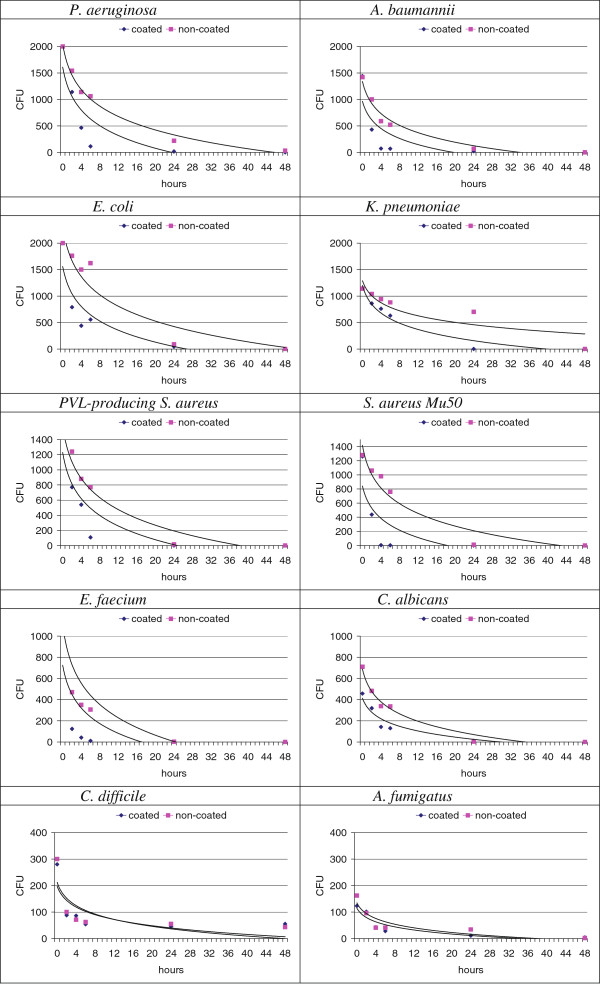
**Schematic representation of the antimicrobial activity of coated and non-coated surfaces contaminated with microorganisms.** Colony counts in average cfu obtained from wires 0 − 48 hours after contamination with various microorganisms. Data are from five replicate experiments.

The data obtained for the contaminated coated and non-coated leadwire sections are reported in Table 1. Examples of plate cultures obtained from Mu50 *S*. *aureus*-coated leadwires are shown in Figure 1. The average colony counts obtained for the non-coated control wires after they were incubated for 2 − 48 hours showed that all the microorganisms tested were able to survive on the surface of the non-coated wires for prolonged periods of time (Table 1) (Figure 2). Culture of these wires six hours after contamination revealed high bacteria counts in all cases. At 24 hours, very high bacteria counts were detected for Gram-negative organisms. At 48 hours, substantial numbers of bacteria colonies were cultured from wires contaminated with most of the Gram-negative microorganisms (i.e. the ESBL-producing *Enterobacteriaceae* and *P*. *aeruginosa*) and with the two spore-forming microorganisms, *A*. *fumigatus* and *C*. *difficile*. In contrast, we detected survival of the two staphylococci, *E*. *faecium* and *C*. *albicans* 24 hours after contamination but never after 48 hours. Overall, these first data confirmed the non-coated leadwire surface as a stand for nosocomial pathogens, capable of harboring viable microorganisms. 

**Table 1:** Colony counts obtained from coated and non-coated surfaces contaminated with microorganisms for 0 − 48 hours

**Microorganisms**	**Colony counts in average cfu (standard deviation) obtained from wires 0 − 48 hours after contamination with various microorganisms**^**1**^**. Data are from five replicate experiments.**							***P***^**2**^
	**Wires**	**0 hr**	**2 hr**	**4 hr**	**6 hr**	**24 hr**	**48 hr**	
*Pseudomonas aeruginosa*	coated	> 2000 (nc)	1140 (89)	464 (42)	114 (60)	17 (12)	2 (1)	
	non-coated	> 2000 (nc)	1540 (89)	1140 (167)	1060 (89)	220 (81)	33 (21)	< 0.001
*Acinetobacter baumannii*	coated	1440 (89)	430 (110)	72 (38)	69 (53)	21 (30)	< 1 (nc)	
	non-coated	1420 (84)	1000 (0)	590 (55)	520 (45)	67 (49)	< 1 (nc)	< 0.001
*Escherichia coli*	coated	> 2000 (nc)	790 (55)	440 (47)	556 (38)	46 (8)	< 1 (nc)	
	non-coated	> 2000 (nc)	1760 (152)	1500 (0)	1620 (164)	90 (70)	2 (1)	< 0.001
*Klebsiella pneumoniae*	coated	1140 (nc)	860 (89)	760 (42)	630 (60)	< 1 (nc)	< 1 (nc)	
	non-coated	1140 (114)	1040 (55)	940 (55)	880 (45)	700 (71)	2 (2)	< 0.001
*Enterobacter cloacae*	coated	> 2000 (nc)	1940 (89)	1500 (0)	1020 (45)	810 (22)	2 (1)	
	non-coated	> 2000 (nc)	> 2000 (nc)	> 2000 (nc)	1480 (45)	569 (321)	2 (2)	0.031
*Mu50 Staphylococcus aureus*	coated	1260 (55)	438 (36)	5 (2)	3 (7)	< 1 (nc)	< 1 (nc)	
	non-coated	1280 (84)	1060 (55)	980 (45)	760 (55)	11 (8)	< 1 (nc)	< 0.001
*PVL Staphylococcus aureus*	coated	> 1500 (nc)	770 (45)	540 (89)	108 (16)	< 1 (nc)	< 1 (nc)	
	non-coated	> 1500 (nc)	1240 (55)	880 (27)	770 (45)	15 (12)	< 1 (nc)	< 0.001
*Enterococcus faecium*	coated	1220 (110)	124 (33)	42 (26)	11 (5)	< 1 (nc)	< 1 (nc)	
	non-coated	1480 (45)	470 (76)	350 (35)	306 (26)	3 (2)	< 1 (nc)	< 0.001
*Clostridium difficile*	coated	280 (45)	88 (12)	86 (15)	54 (15)	46 (12)	55 (22)	
	non-coated	300 (0)	100 (17)	71 (10)	62 (15)	55 (4)	43 (9)	0.328
*Candida albicans*	coated	458 (62)	319 (23)	143 (38)	130 (25)	11 (7)	< 1 (nc)	
	non-coated	710 (86)	482 (63)	338 (24)	336 (32)	4 (4)	< 1 (nc)	< 0.001
*Aspergillus fumigatus*	coated	123 (22)	101 (9)	43 (12)	28 (7)	11 (4)	4 (4)	
	non-coated	162 (44)	96 (18)	41 (9)	41 (9)	34 (5)	2 (1)	0.061

*Nc*: not calculable; ^1^at t0 and after 2, 4, 6, 24 or 48 hours of storage, contaminated leadwire sections were rolled on blood agar plates. Plate colonies were counted after incubation for 48 hours at 37°C; ^2^Colony counts were compared using the Wilcoxon signed-rank test to determine statistical significance.

In contrast, the coated surfaces exhibited marked biocidal activity, relative to that of the control surface, against all non-spore-forming microorganisms tested (Table 1). The biocidal effect of the coated surface differed with respect to the microorganisms in terms of kinetics, intensity and final outcome. Nevertheless, bacterial counts after six to 24 hours of contact with the coated surface were significantly lower than those observed with non-coated surface (p <0.001). The biocidal effect was the fastest against staphylococci and *E*. *faecium*, as marked effects were detected after two hours of contact. In contrast, the biocidal effect on Gram-negative microorganisms took longer and was generally evident after four hours. Of note, the spore-forming microorganisms were completely unaffected by the coated surfaces.

The acidic surface reaction is thought to be related to the diffusion of H_3_O^+^ ions through the microbial membranes, resulting in altered enzymatic transport systems and inhibition of key metabolic activities [17]. Thus, the acidic-pH-associated biocidal effect produced by the metalloacid-coated surface would be expected to have a universal effect. The variations in susceptibility between Gram-positive and Gram-negative microorganisms, and the resistance exhibited by the spore-forming ones, suggest that the differences among microorganisms with respect to their susceptibility to the biocidal surface may be due to the H_3_O^+^ ion permeability of their cell wall and/or cell membrane. In account of a biological cost of antimicrobial resistance, MDR microorganisms may be less fit and have slower growth rates than sensitive strains [19]. Thus, further studies should be done with wild isolates, to guaranty a biocide effect on them. Despite these limitations, our data show that the survival of non-spore-forming multidrug resistant microorganisms was severely diminished on coated surfaces compared to that on control surfaces. Considering the continuous spread of microorganisms in the surroundings of patients, the usual rhythm of cleaning procedures in hospital wards, and the continuous biocidal effect of the metalloacid-coated surface against the multidrug resistant nosocomial pathogens, we suggest that coated device surfaces may provide an permanent means of minimizing microbial contamination between two cleaning procedures.

The mechanism of microorganism killing at pH values <4.0 is non-specific [1]. Thus, in contrast to disinfectants and antibiotics, microbial resistance to this mode of action may not emerge as a result of exposure to MoO_3_. In addition, cytotoxicity tests (MTT assays) and thrombogenicity tests of the powder components suggest that they should be safe for human use [16].

## Conclusions

In view of these promising *in vitro* results, we suggest that MoO_3_ coating may be an effective means of minimizing microorganism contamination on certain hospital surfaces. Further studies are needed to evaluate the benefits of this coating on medical devices that are frequently touched by HCWs and to determine whether it can be used as a complementary measure for the preventing of the spread of microorganisms to sites near patients in hospital settings.

## Competing interests

The authors declare that they have no competing interests.

## Authors’ contributions

NVDM conceived the study and wrote the manuscript. NT carried out the microbiological tests. HGH conducted the statistical analysis. XB and RQ participated in the design of the study and helped draft the manuscript. All authors read and approved the final manuscript.
